# The trade-off of post-mastectomy radiotherapy usage for the breast cancer patients aged 70 years or older: a study based on SEER database

**DOI:** 10.1186/s12877-023-04341-y

**Published:** 2023-10-06

**Authors:** Jingyi Lin, Shiping Luo, Jie Zhang, Chuangui Song

**Affiliations:** 1 Department of Breast Surgery, Fujian Medical University Union Hospital, No.29, Xin Quan Road, Gulou District, Fuzhou, Fujian Province 350001 China; 2 Department of General Surgery, Fujian Medical University Union Hospital, Fuzhou, Fujian Province 350001 China; 3 Breast Cancer Institute, Fujian Medical University, Fuzhou, Fujian Province 350001 China

**Keywords:** Postmastectomy radiotherapy, Elderly patients, Propensity score matching, Nomogram, SEER

## Abstract

**Background:**

This study aimed to investigate the role of post-mastectomy radiotherapy (PMRT) in the female aged 70 years or older diagnosed with breast cancer, which is still controversial.

**Methods:**

This retrospective study enrolled female breast cancer women aged 70 + years following mastectomy from the Surveillance, Epidemiology, and End Results (SEER) database. Propensity score matching (PSM) was performed to reduce covariable imbalance. A nomogram was created to predict the 1,3,5-years overall survival (OS) and divide patients into three risk groups.

**Results:**

After matching, PMRT were associated with significant improvement in breast cancer-specific survival (BCSS) and OS (p < 0.001). By contrast, the BCSS and OS benefit from PMRT were not significant in patients with T1N1 tumor (BCSS: HR = 0.716, p = 0.249;OS:HR = 0.908, p = 0.572), and T2N1 tumor (BCSS:HR = 0.866, p = 0.289;OS:HR = 0.879, p = 0.166). Stratified by subtype, the HR+/HER-2- subtype and the HR-/HER-2- subtype (all p < 0.001) have a significant prolonged survival, yet not significant BCSS difference are shown in the HER-2 + tumor. In the low-risk group as determined by the nomogram, the use of PMRT did not significantly improve OS (p = 0.203).

**Conclusions:**

This study demonstrated that PMRT improves the survival of females with elderly breast cancer, while for T1-2N1 breast cancer patients, the omission of PMRT could be considered. Furthermore, the nomogram we constructed could be used as a decision tool for the omission of PMRT in low-risk elderly patients.

**Supplementary Information:**

The online version contains supplementary material available at 10.1186/s12877-023-04341-y.

## Introduction

As the most common female malignancy globally, breast cancer accounts for nearly 30% of all tumors diagnosed in female patients [1]. With the worldwide population aging gradually, the burden of tumors continues to increase, and the elderly malignancy grows to be a non-negligible health issue worldwide. It is reported that almost half of breast cancer-specific deaths occur in female patients aged 70 or over [2]. A 2019 study about global cancer incidence estimated that the number of newly diagnosed cancer in the elderly population would be double by 2035, from 2.8 to 5.7 million among elderly females. By 2035, 58% of the total cancer incidence globally will happen in older people [3].

As a primary treatment of local therapy, the appropriate usage of radiotherapy can reduce the local recurrence rate and prolong survival. In a pity, limited category I evidence to date has demonstrated the role of postmastectomy radiotherapy in the cohort of elderly female patients [4]. The majority of recommendations are composed of extrapolation from analyses in all-age patients [5]. NCCN guidelines supported postmastectomy radiotherapy for patients with 4 or more positive lymph nodes, and “strongly recommended” for those with 1–3 positive lymph nodes. Although multiple cancer care guidelines recommending its usage had been published, no improved usage rate in PMRT was observed between 1999 and 2005. Indeed, only 54.8% of high-risk (T3/T4 and/or N2/N3) patients received PMRT [6].

It was considered in the past that PMRT was associated with some long-term side effects, such as cardiovascular system diseases, secondary cancer, and arm lymphedema, which shouldn’t be ignored. However, with the advancement of radiotherapy techniques, the death risk from side effects caused by PMRT has significantly decreased over time [7]. On the other hand, in the era of increasingly effective comprehensive systematic therapy, the status of PMRT use is being challenged [9]. Based on the above considerations, the impact of PMRT on elderly breast cancer patients should be reassessed, especially for low-risk patients.

This study used data from Surveillance, Epidemiology, and End Results Program (SEER) database, aiming to investigate the role of post-mastectomy radiotherapy in females aged 70 years or older diagnosed with breast cancer. On this basis, we further constructed a nomogram to predict the prognosis of elderly breast cancer patients for the sake of identifying the population who could omit PMRT safely.

## METHODS

### Population

**Fig. 1:**
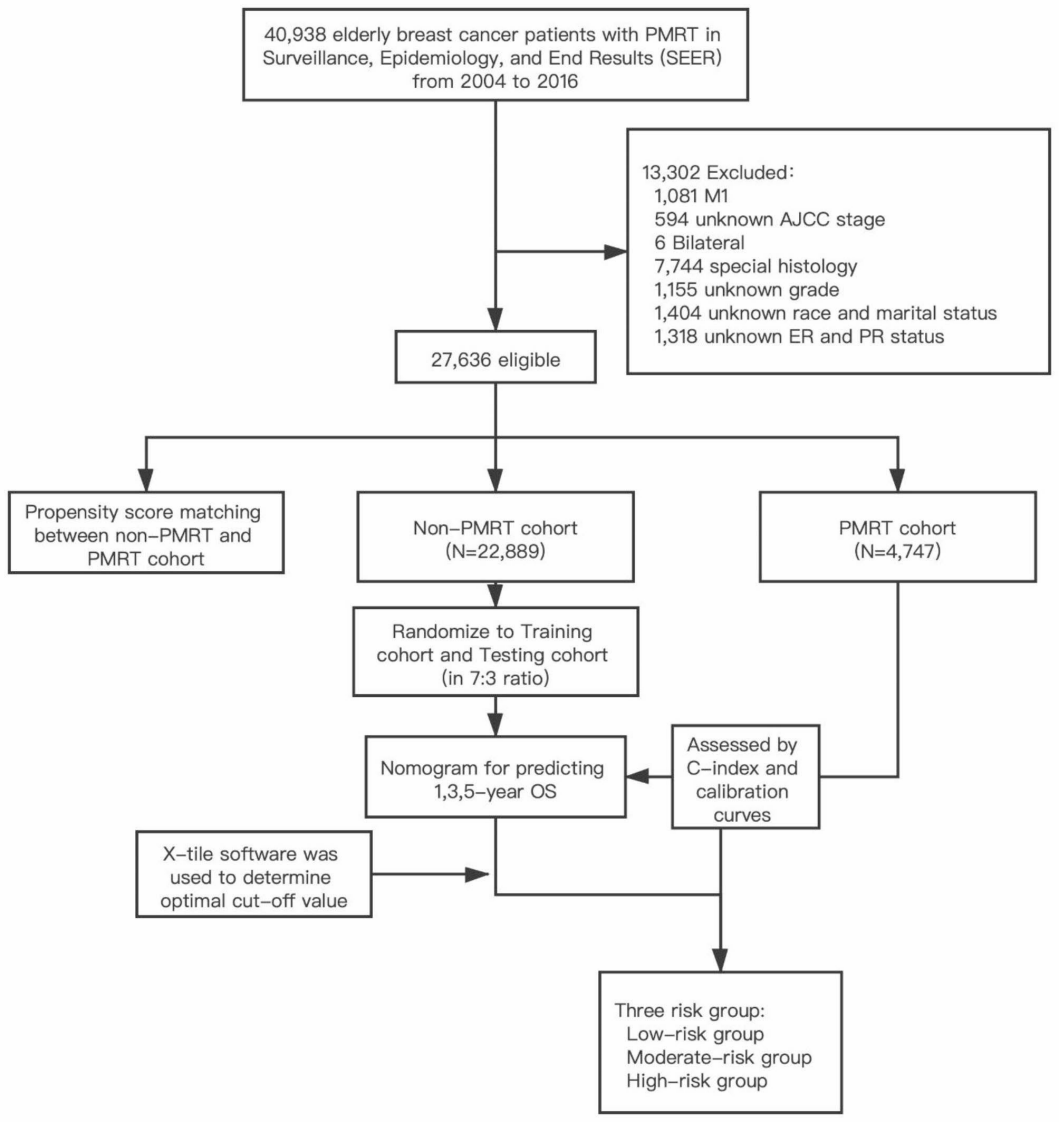
Flowchart of this study design. PMRT: post-mastectomy radiotherapy; ER: estrogen receptor; PR: progesterone receptor

The flowchart of this study design is shown in Fig. 1. Women aged 70 years or more diagnosed with breast cancer between 2004 and 2016 following mastectomy extracted from the Surveillance, Epidemiology, and End Results Program (SEER) database met the inclusion criteria. The exclusion criteria are as follows: M1, bilateral, special histological types, multiple primary carcinomas, unknown histology grade, autopsy or death certificate only, pre-and intraoperative radiation, unknown ER/PR status, unknown marital status and race, and unknown examined lymph nodes number. Data included in the calculations contains age at diagnosis, race, marital status, laterality, histology grade, AJCC T stage, AJCC N stage, chemotherapy status, lymph node surgery status, ER and PR status, HER-2/neu status, and subtype.

### Statistical analysis

Based on with or without PMRT, patients were divided into PMRT cohort and non-PMRT cohort. In the survival analysis, breast cancer-specific survival (BCSS) was defined as the time from diagnosis until death from breast cancer, overall survival (OS) was defined as the time from first diagnosis to all-cause death or the date of the last follow-up. Propensity score matching (PSM) was applied to control confounding factors such as selection bias to make two cohorts comparable. The difference in baseline clinicopathological characteristics before and after PSM were compared by Chi-square and Fisher’s exact probability tests. After PSM, standardized mean difference (SMD) < 10% was considered a sufficient balance criterion [11]. We performed Kaplan-Meier survival analyses combined with log-rank tests to determine whether the differences in BCSS or OS between patients receiving adjuvant radiotherapy and those not receiving it were statistically significant. We calculated hazard ratios (HR) with 95% confidence intervals (CI) using univariate and multivariate Cox proportional hazard regression models.

In the sensitivity analysis, we categorized causes of death into the breast cancer-specific death (BCSD) group and the non-BCSD group. Gray’s test was employed to assess the statistical differences between BCSD and non-BCSD events, considering the presence of competing risk events. The subdistribution hazard ratio (SHR) for BCSD was estimated using the Fine and Gray competing risk model, taking into account the variables under consideration.

To intuitively explain the impact of prognostic factors on survival for the patient in the non-PMRT cohort, we built a nomogram. Patients in the non-PMRT cohort were randomized into a training cohort and a validation cohort in a 7:3 ratio. The univariable and multivariable COX regression model assessed independent prognostic variables that could influence outcomes. Accordingly, we create a nomogram to predict the 1,3,5-years OS. The discrimination of the nomogram was evaluated using the concordance index (C-index), and calibration curves were formulated to assess the consistency between predicted and actual outcomes. The X-tile software determined the optimal cutoff value of risk scores from the nomogram and divided patients into three groups (low risk, moderate risk, and high risk) [12]. All statistical analyses were performed by R statistical software (version 4.0.3, http://www.R-project.org/.) and SPSS (version 22.0).

## Results

### Clinicopathological characteristics

Of the 27,636 women met all criteria for inclusion and exclusion, 17.2% (n = 4,747) received PMRT while 82,8% (n = 22,889) not. The median follow-up time was 73 months. Clinicopathological characteristics among the two arms are presented in Table 1. Overall, RT-received patients have more invasive characteristics like younger, higher histology grades, larger tumor size, more No. of positive lymph nodes, and negative hormone receptor status. There was a significant reduction of adjuvant radiotherapy utilization in older patients, from 19.7% of the 70–74 years group to 12.6% of 85 years or older group. A distribution difference in PMRT usage also occurred among hormone receptor status groups. ER- group and PR- group seems to tend to receive PMRT, with a PMRT usage of 18.3% and 18.5%, respectively. In contrast, ER + group and PR + group had less usage of 16.9% and 16.5%, respectively. Additionally, patients with chemotherapy treatment occupy a less proportion of non-PMRT arm, which received versus not received chemotherapy were 17.4% versus 82.6%. In contrast, that proportion in the PMRT arm was similar (51.3% versus 48.7%).

**Table 1:** Baseline characteristics of elderly patients with breast cancer before and after PSM

Characteristics	Before PSM			After PSM			
	Non-PMRT (N = 22,889)	PMRT (N = 4747)	P value	Non-PMRT (N = 3777)	PMRT (N = 3777)	P value	SMD
Age group at diagnosis, y			< 0.001			0.586	0.032
70–74	8028 (35.1)	1970 (41.5)		1476 (39.1)	1527 (40.4)		
75–79	6634 (29.0)	1406 (29.6)		1123 (29.7)	1079 (28.6)		
80–84	4869 (21.3)	883 (18.6)		745 (19.7)	731 (19.4)		
85+	3358 (14.7)	488 (10.3)		433 (11.5)	440 (11.6)		
Race			0.156			0.637	0.022
White	18,868 (82.4)	3906 (82.3)		3183 (84.3)	3154 (83.5)		
Black	2054 (9.0)	460 (9.7)		346 ( 9.2)	358 (9.5)		
Others	1967 (8.6)	381 (8.0)		248 ( 6.6)	265 (7.0)		
Marital Status			< 0.001			0.981	0.001
Married	9252 (40.4)	2057 (43.3)		1547 (41.0)	1549 (41.0)		
Unmarried	13,637 (59.6)	2690 (56.7)		2230 (59.0)	2228 (59.0)		
Laterality			0.874			0.982	0.001
Left	11,821 (51.6)	2445 (51.5)		1959 (51.9)	1957 (51.8)		
Right	11,068 (48.4)	2302 (48.5)		1818 (48.1)	1820 (48.2)		
Grade			< 0.001			0.988	0.004
Grade I	4340 (19.0)	543 (11.4)		399 (10.6)	402 (10.6)		
Grade II	10,491 (45.8)	2108 (44.4)		1694 (44.9)	1697 (44.9)		
Grade III + IV	8058 (35.2)	2096 (44.2)		1684 (44.6)	1678 (44.4)		
T			< 0.001			0.591	0.032
T0/T1	11,500 (50.2)	866 (18.2)		735 (19.5)	746 (19.8)		
T2	9134 (39.9)	2197 (46.3)		1902 (50.4)	1859 (49.2)		
T3	1395 (6.1)	1036 (21.8)		749 (19.8)	748 (19.8)		
T4	860 (3.8)	648 (13.7)		391 (10.4)	424 (11.2)		
N			< 0.001			0.506	0.035
N0	14,966 (65.4)	975 (20.5)		899 (23.8)	943 (25.0)		
N1	5659 (24.7)	1685 (35.5)		1514 (40.1)	1465 (38.8)		
N2	1460 (6.4)	1347 (28.4)		882 (23.4)	868 (23.0)		
N3	804 (3.5)	740 (15.6)		482 (12.8)	501 (13.3)		
ER			0.017			0.909	0.003
Negative	4462 (19.5)	998 (21.0)		776 (20.5)	771 (20.4)		
Positive/Borderline	18,427 (80.5)	3749 (79.0)		3001 (79.5)	3006 (79.6)		
PR			< 0.001			0.262	0.026
Negative	7549 (33.0)	1719 (36.2)		1365 (36.1)	1413 (37.4)		
Positive/Borderline	15,340 (67.0)	3028 (63.8)		2412 (63.9)	2364 (62.6)		
Chemotherapy			< 0.001			0.531	0.015
No/Unknown	18,902 (82.6)	2311 (48.7)		2157 (57.1)	2129 (56.4)		
Yes	3987 (17.4)	2436 (51.3)		1620 (42.9)	1648 (43.6)		
LN Surgery						0.145	0.034
SLNB	11,571 (50.6)	1198 (25.2)	< 0.001	1036 (27.4)	1094 (29.0)		
ALND	11,318 (49.4)	3549 (74.8)		2741 (72.6)	2683 (71.0)		

PMRT = post-mastectomy radiotherapy; ER = estrogen receptor; PR = progesterone receptor; PSM = propensity score matching; SMD = standardized mean difference;

LN = lymph node; SLNB = sentinel lymph node biopsy; ALND = axillary lymph node dissection

### Propensity score matching and subgroup analysis

**Fig. 2:**
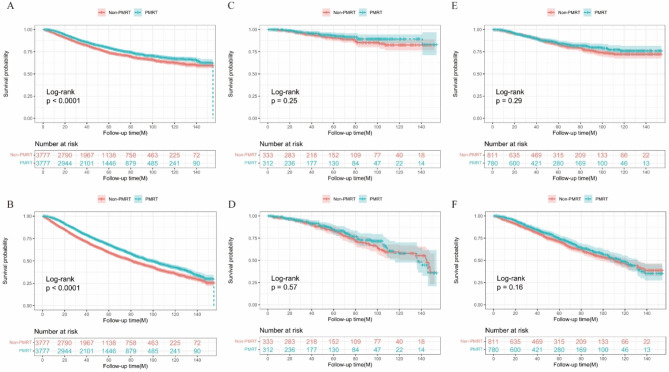
Kaplan-Meier curves for comparing the BCSS and OS of propenstiy-score matched patients with or without PMRT. (**A**) BCSS and (**B**) OS in the whole cohort; (**C**) BCSS and (**D**) OS in the T1N1 tumor group; (**E**) BCSS and (**F**) OS in the T2N1 tumor group. BCSS: Breast cause-specific survival; OS: Overall survival; PMRT: Postmastectomy radiotherapy

After propensity score matching in a 1:1 ratio, there were 3,777 patients in each arm, and the clinicopathological characteristics across two arms were well-balanced (all SMD < 10%, Table 1). In whole cohort after PSM, as shown in Fig. 2A-B, PMRT was associated with significant improvement in terms of BCSS (HR = 0.790, 95%CI 0.715–0.874, p < 0.001) and OS (HR = 0.755, 95%CI 0.701–0.813, p < 0.001).

**Fig. 3:**
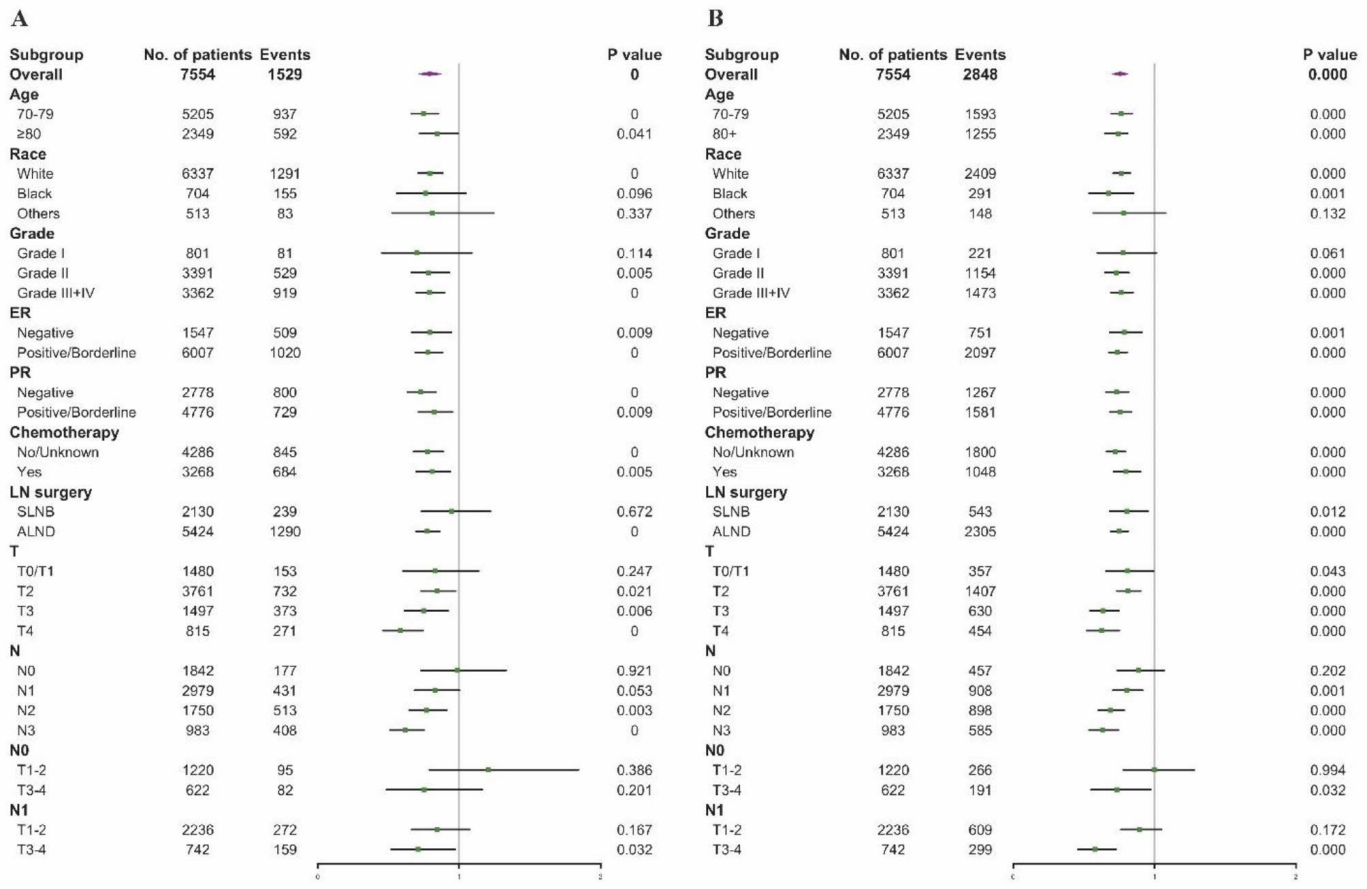
Forest plots of multivariable COX regression analysis for (**A**) BCSS and (**B**) OS in matched patients. PMRT: post-mastectomy radiotherapy; ER: estrogen receptor; PR: progesterone receptor; LN: lymph node; SLNB = sentinel lymph node biopsy; ALND = axillary lymph node dissection

To further identify patients who may benefit from PMRT, subgroup analyses were performed on the basis of clinicopathological factors, especially tumor size, number of positive lymph nodes, and hormone receptor status. Figure 3 demonstrates that the BCSS and OS benefit from PMRT was observed in most subgroups. Except for patients with Grade I tumor, those of Other races, and those with negative lymph nodes, survival between those with and without PMRT didn’t show the difference (all p > 0.05). Additionally, there was no observed improvement in BCSS for Black patients, those with T0/T1 stage, N1 stage, and those who underwent SLNB (all p > 0.05).

Based on these above findings, patients were further divided into subgroups according to tumor size and the number of positive lymph nodes (shown in Fig. 2) to investigate the possible omission of PMRT in specific populations. Results show that patients with T1N1 tumor could not get the benefit from PMRT, and survival among the two groups was similar (BCSS: HR = 0.716, 95%CI 0.406–1.263, p = 0.249; OS: HR = 0.908, 95%CI 0.648–1.270, p = 0.572, Fig. 2C-D). The same findings were found in patients with T2N1 tumor, whose survival did not show a significant difference (BCSS: HR = 0.866, 95%CI 0.664–1.130, p = 0.289; OS: HR = 0.879, 95%CI 0.732–1.055, p = 0.166, Fig. 2E-F).

**Fig. 4:**
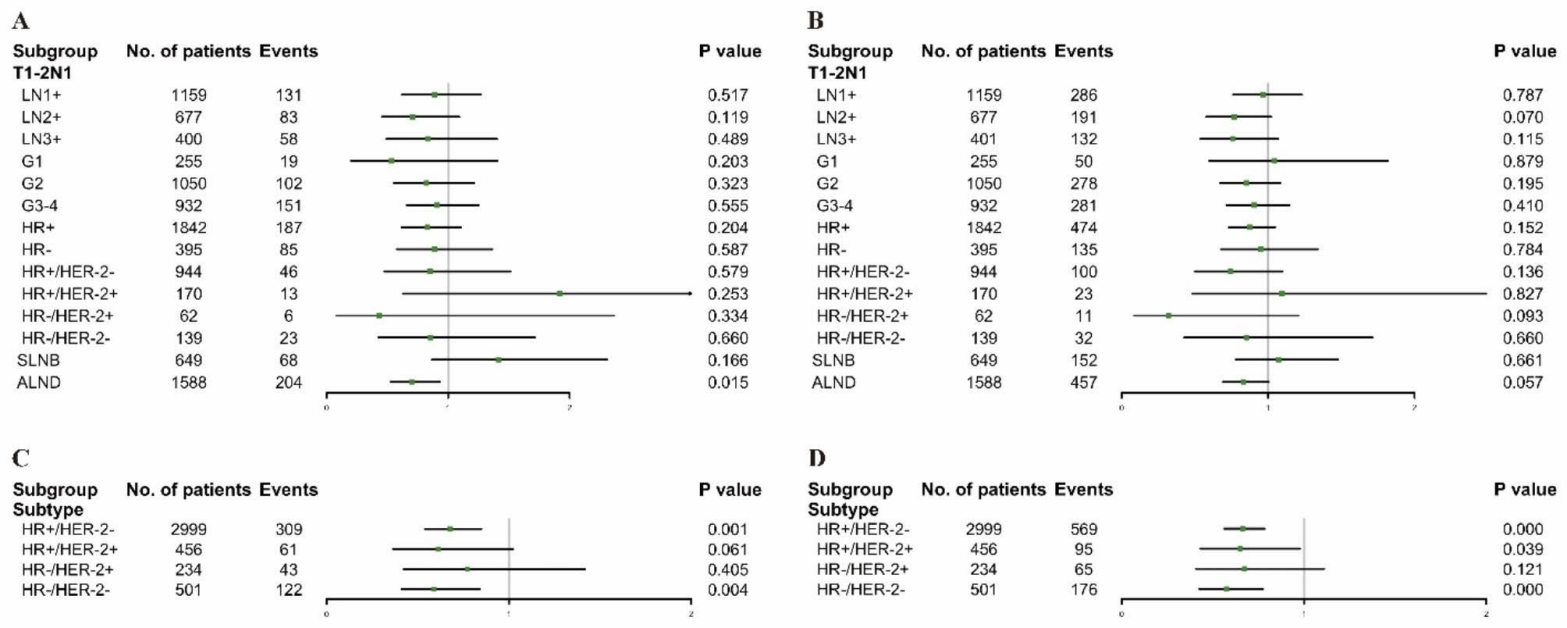
Forest plots of multivariable COX regression analysis for (**A**) BCSS and (**B**) OS in matched patients with T1-2N1 breast cancer; Forest plots of multivariable COX regression analysis for (**C**) BCSS and (**D**) OS in patients rematched by known subtype. LN: lymph node; G: Grade; HR: hormone receptor; HER-2: human epidermal growth factor receptor 2; SLNB = sentinel lymph node biopsy; ALND = axillary lymph node dissection

Then in further subdividing T1-2N1 tumor according to positive numbers of lymph nodes, HR status, grade, subtype, and lymph node surgery, shown in Fig. 4, we found that PMRT did not improve survival for most subgroups, consistent with the above findings. Only among patients who underwent ALND did PMRT statistically improve the BCSS (p = 0.015).

To illustrate the prognostic value of subtype, we conducted a rematch in a 1:1 ratio by adjustment for age at diagnosis, race, marital status, grade, T and N classification, chemotherapy, and subtype. As shown in Fig. 4, there are significant improvement on BCSS and OS of PMRT for the HR+/HER-2- subtype (BCSS: p = 0.001; OS: p < 0.001), and the HR-/HER-2- subtype (BCSS: p = 0.004; OS: p < 0.001). For the HR+/HER-2 + subtype, no association was observed between BCSS and radiotherapy, and the difference was not statistically significant (p = 0.061). However, an improvement in OS was observed in this subgroup (p = 0.039). But no BCSS and OS difference were observed in the HR-/HER-2 + subtype (BCSS: p = 0.405; OS: p = 0.121).

### Sensitivity analysis

**Fig. 5:**
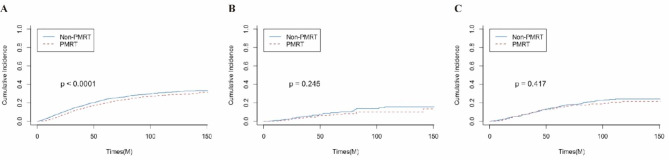
Cumulative incidence of breast cancer-specific mortality in (**A**) all patients, (**B**) T1N1 patients, and (**C**) T2N1 patients, stratified by receipt of postmastectomy radiation therapy (PMRT)

Sensitivity analyses were conducted to explore the impact on survival outcomes. Consistent with the previous analysis, the results showed that (Fig. 5) in the matched overall population, patients who underwent PMRT demonstrated a significantly lower breast cancer-specific death rate compared to those who did not receive PMRT (p < 0.0001). However, in the T1N1 and T2N1 subgroups, PMRT did not show a significant effect on the breast cancer-specific death rate (T1N1, p = 0.245; T2N1, p = 0.417). Supplementary Fig. 1 present the results of the Fine and Gray model, where no significant survival improvement was observed in patients aged 80 years or older, those with T0/T1 stage tumors, Grade I tumors, individuals of Black or Other races, those with negative lymph nodes, PR-positive tumors, and those who underwent SLNB (all p > 0.05).

Furthermore, upon further subgroup analysis of T1-2N1 tumors (Supplementary Fig. 2A) or re-matching patients based on subtypes (Supplementary Fig. 2B), the results remained consistent with the Cox analysis.

### Establishment of a prognostic nomogram

**Fig. 6:**
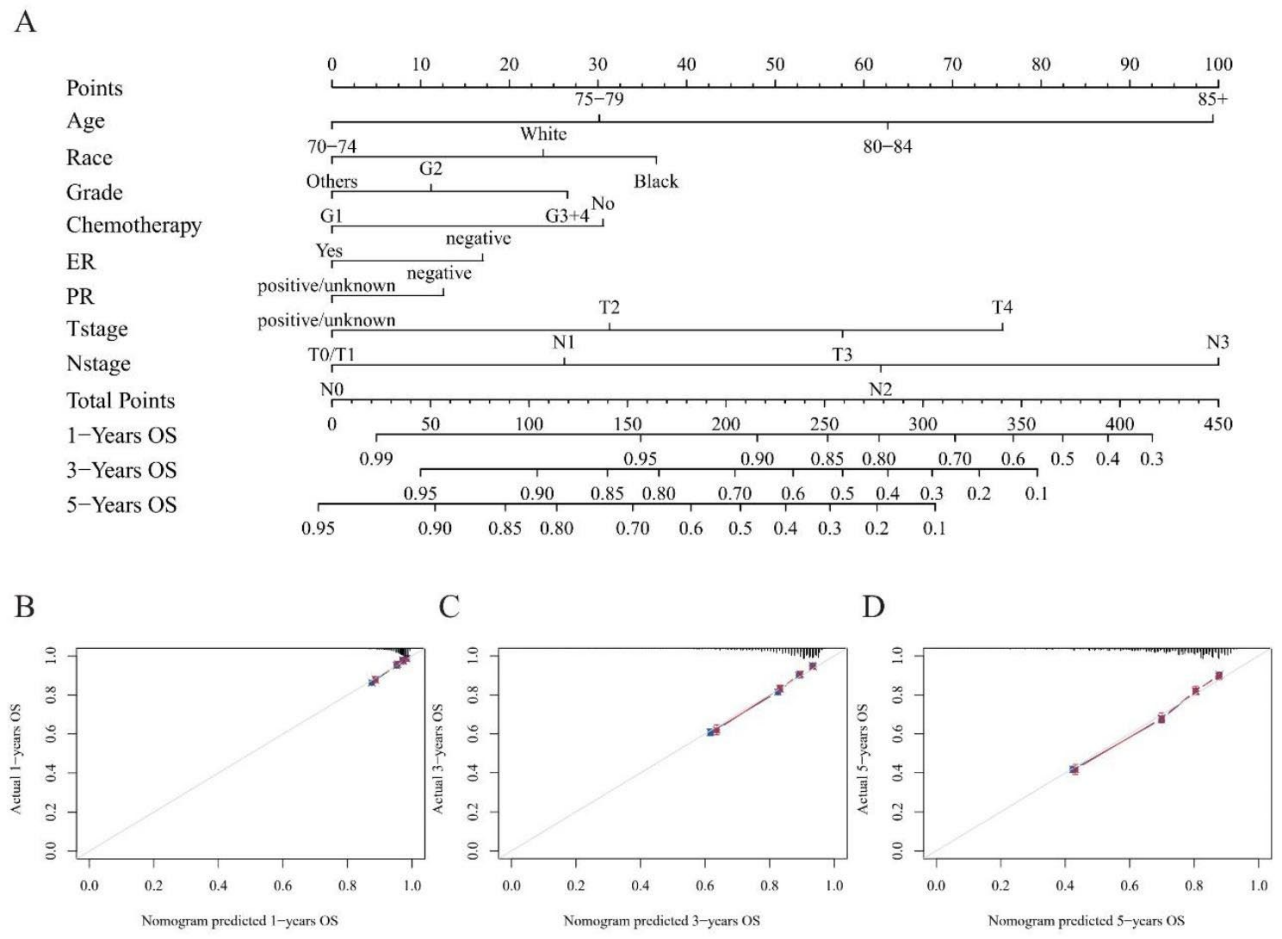
(**A**) Nomogram and its calibration curves of (**B**) 1-, (**C**) 3-, (**D**) 5-years OS for non-PMRT patients. Blue line: calibration curves in the training cohort; Red line: calibration curves in the validation cohort. ER: estrogen receptor; PR: progesterone receptor; OS: Overall survival

We performed the univariable and multivariable analysis of the non-PMRT cohort. The results are shown in Table 2. Age, race, marital status, histology grade, T stage, N stage, ER status, PR status, and given chemotherapy were independent prognostic factors for the overall survival of elderly breast cancer patients in the non-PMRT cohort (all p < 0.001). Next, 22,889 patients in the non-PMRT cohort were randomized into the training cohort and the validation cohort in a 7:3 ratio. A nomogram was created by the above independent prognostic factors to explain the impact of prognostic factors on survival intuitively and to predict patients’ 1-year, 3-year, and 5-year OS (Fig. 6). The prediction nomogram had an acceptable discrimination capacity for distinguishing between alive and dead patients (C-index was 0.72). The 1-year, 3-year, and 5-year calibration curves demonstrated excellent agreement across prediction and actual observation in the training and validation cohorts (Fig. 6).

**Table 2:** Prognostic factors for OS in elderly patients without PMRT by univariable and multivariable analysis

Characteristics	Univariable			Multivariable		
	Hazard ratio	95%CI	*P* value	Hazard ratio	95%CI	*P* value
Age group at diagnosis, y			0.000			0.000
70–74	Reference			Reference		
75–79	1.535	1.440–1.637	0.000	1.449	1.359–1.546	0.000
80–84	2.440	2.291-2.600	0.000	2.133	1.998–2.277	0.000
≥85	4.241	3.975–4.525	0.000	3.162	2.949–3.390	0.000
Race			0.000			0.000
White	Reference			Reference		
Black	1.199	1.113–1.290	0.000	1.360	1.236–1.497	0.000
Others	0.656	0.596–0.721	0.000	1.510	1.344–1.697	0.000
Marital Status			0.000			0.000
Married	Reference			Reference		
Unmarried	1.708	1.629–1.791	0.000	1.270	1.209–1.334	0.000
Laterality			0.512			
Left	Reference					
Right	0.986	0.944–1.029	0.512			
Grade			0.000			0.000
Grade I	Reference			Reference		
Grade II	1.325	1.240–1.417	0.000	1.101	1.029–1.178	0.005
Grade III + IV	1.899	1.776–2.030	0.000	1.337	1.243–1.439	0.000
T			0.000			0.000
T0/T1	Reference			Reference		
T2	1.827	1.742–1.917	0.000	1.431	1.360–1.505	0.000
T3	3.151	2.906–3.416	0.000	1.904	1.745–2.077	0.000
T4	4.664	4.260–5.108	0.000	2.343	2.123–2.585	0.000
N			0.000			0.000
N0	Reference			Reference		
N1	1.556	1.479–1.636	0.000	1.381	1.311–1.456	0.000
N2	2.893	2.690–3.110	0.000	2.103	1.945–2.273	0.000
N3	4.649	4.260–5.074	0.000	3.312	3.011–3.642	0.000
ER			0.000			0.000
Negative	Reference			Reference		
Positive/Borderline	0.686	0.652–0.722	0.000	0.817	0.762–0.877	0.000
PR			0.000			0.000
Negative	Reference			Reference		
Positive/Borderline	0.704	0.673–0.736	0.000	0.854	0.805–0.906	0.000
Chemotherapy			0.000			0.000
No/Unknown	Reference			Reference		
Yes	0.803	0.754–0.855	0.000	1.454	1.358–1.558	0.000

PMRT = post-mastectomy radiotherapy; ER = estrogen receptor; PR = progesterone receptor; OS = overall survival

**Fig. 7:**
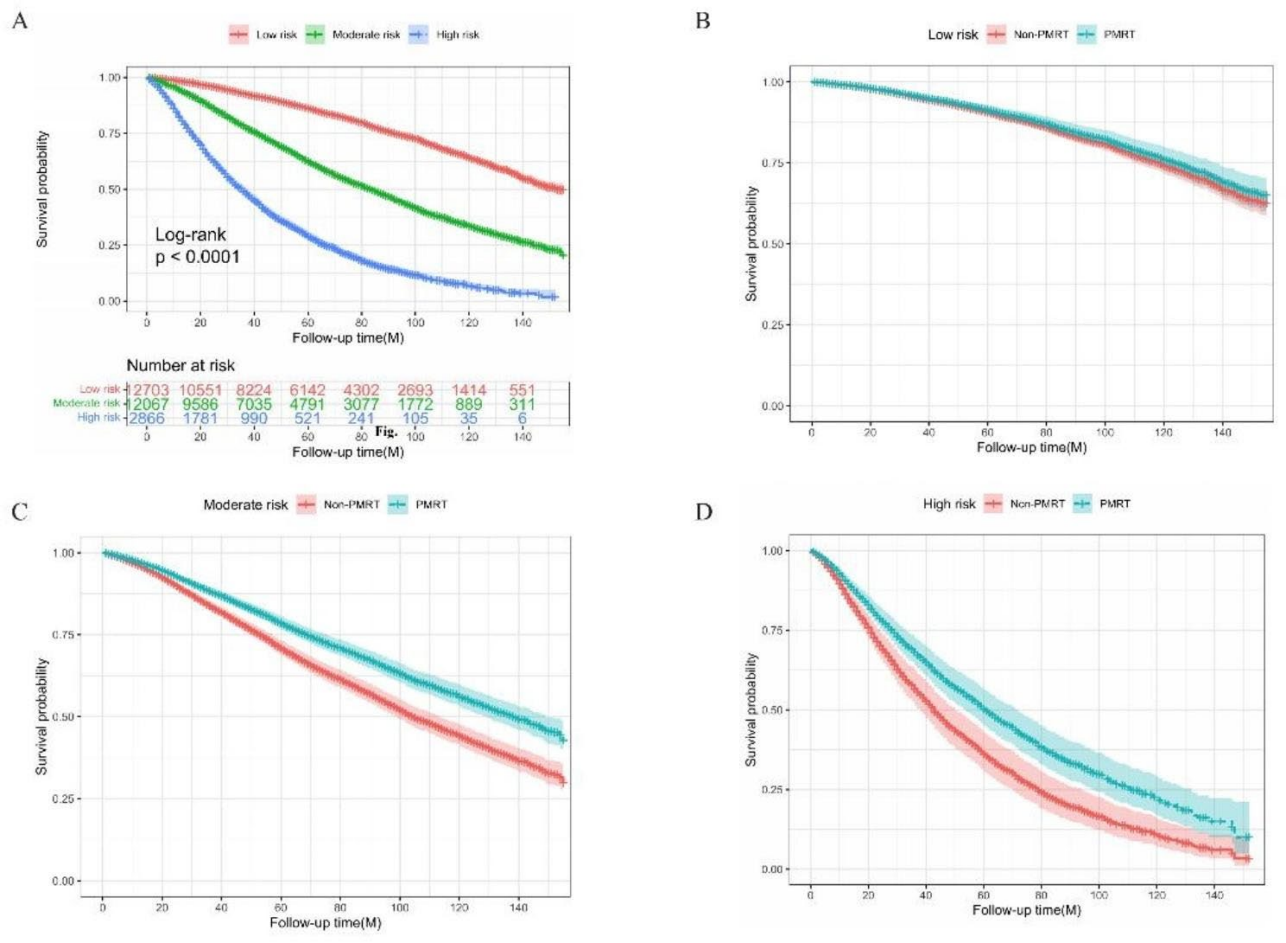
Kaplan-Meier curves for comparing the overall survival of patients in (**A**) three risk groups, (**B**) low-risk group, (**C**) moderate-risk group, and (**D**) high-risk group. These risk groups were divided from the entire cohort by the optimal cut-off values calculated using X-tile. PMRT: post-mastectomy radiotherapy

Furthermore, we calculated the risk scores of all patients according to the nomogram, then identified the optimal cut-off value of scores by X-tile software. Accordingly, 27,636 patients were divided into three groups: 12,703 in the low-risk group (risk score < 140), 12,067 in the moderate-risk group (risk score: 140–241), and 2,866 in the high-risk group (risk score > 241). The Kaplan-Meier curve demonstrates that patients in the low-risk groups have a better prognosis than the other two groups (p < 0.001, Fig. 7). Corrected by Cox regression, PMRT was associated with the improved OS of patients in the moderate-risk and high-risk groups, while no OS difference was observed in the low-risk groups (p = 0.203, Fig. 7), suggesting that this nomogram could sufficiently identify patients who were unable to benefit from PMRT.

## Discussion

To critically assess the impact of PMRT in elderly patients, we conducted a large retrospective population-based study in older women from the SEER database and further analyzed various subgroups seeking to investigate the impact of adjuvant radiotherapy in various subgroups of elderly breast cancer patients.

The rather limited category I evidence has demonstrated the impact of PMRT usage in older women, and there is no randomized controlled trial evaluating PMRT in this population. A comparative study about the SEER database from 1992 to 1999 explored the impact of PMRT on elderly breast cancer. Results have shown that PMRT was not associated with improved survival in the whole cohort (adjusted HR 1.03; P = 0.49), only favor the patients in the high-risk group (T3/T4 and/or N2/N3) [13]. In our study, after adjusting confounding factors by the propensity score matching, PMRT substantially prolongs the breast cancer-specific and overall survival of elderly patients, especially with unfavorable disease features as T3-T4 and/ or N2-N3 stage. It demonstrated that PMRT still improves the outcomes of most elderly patients.

To date, adjuvant radiotherapy for patients with less than four positive lymph nodes has not yet become a consensus. Previous studies, such as the British Columbia randomized trials, the Danish Breast Cancer Cooperative Group (DBCG) protocols 82b and 82c trials, showed the significant improvement of adjuvant radiotherapy on survival [14]. A large meta-analysis conducted by the Early Breast Cancer Trialists Collaborative Group (EBCTCG) demonstrated that PMRT could reduce locoregional recurrence, overall recurrence, and breast cancer mortality for women with axillary dissection and 1–3 positive lymph nodes, even when stratified by age [17]. In contrast, at the retrospective analysis of the SEER database (1992–1999), for low-risk (T1/T2 N0) and intermediate-risk (T1/T2 N1) breast cancer, PMRT did not improve survival. While for high-risk (T3/T4 and/or N2/N3) patients, PMRT was associated with a significant improvement in survival (p = 0.02) [13]. However, the EBCTCG analysis evaluated trials that recruited patients from 1964 to 1986, and the retrospective analysis of the SEER database involved patients from 1992 to 1999. This period’s systemic therapy and RT treatment differed significantly from the new therapies used in the modern treatment era. Also, the analysis in EBCTCG did not focus on patients with less than 5 cm size tumors. Therefore, reevaluating survival outcomes in elderly patients impacted by PMRT is warranted, particularly for those with T1-2N1.

Our study found that there was no significant correlation between PMRT and survival for patients with T1-2N1 tumors, both on BCSS and OS. This finding was consistent with the further analysis of patients with T1-2N1 tumors stratified by different grades, different numbers of positive LNs, different HR statuses, and different subtypes. In accordance with our findings, Patel et al. investigated the role of PMRT in the treatment of patients with T1-T2, N1mic disease, and their results showed that PMRT does not affect the survival of breast cancer patients with N1mic disease, even though elderly patients only accounted for a small portion of the population (with 16% of patients being 65 years or older) [18]. Contrasting our study, a previous study published by Zhou et al. found that PMRT could improve OS for patients aged 75 + years old with a tumor size of ≤ 5 cm and 1–3 positive lymph nodes [19]. Whereas it is a pity that the population in that study consisted of earlier diagnosed patients (between 1998 and 2005), and the missing data of chemotherapy therapy also could affect the reliability of this study’s results. In addition, another recent clinical trial reported by Cao et al. showed that PMRT could not improve the survival outcome for all elderly patients with 1–3 positive lymph nodes. Only an improvement in survival by PMRT was detected in patients with tumors > 5 cm, [20]. which is supportive of our results. Combined with these results, our findings further confirm the feasibility of separating patients with 5 cm less or above tumor in the discussion of PMRT usage range.

In our study, PMRT did not improve BCSS and OS for patients with 1, 2, or 3 positive lymph nodes. Luo et al [21] also discussed this topic, but there are some differences in their findings compared to ours. In their retrospective analysis of the SEER database, they specifically assessed the impact of radiotherapy on different numbers of positive lymph nodes and found that only patients with tumor diameters of 2–5 cm, 3 positive lymph nodes, and no chemotherapy received survival benefits from PMRT. The limitation is that this study did not achieve a good balance in terms of age and tumor stage between the radiotherapy group and the non-radiotherapy group, which may have led to the differences in findings compared to our study, considering that these factors are significantly associated with survival outcomes. Furthermore, Chen et al [22] analyzed breast cancer patients aged 75 and older with T3 and lymph node-negative disease, finding that those who received adjuvant radiotherapy showed a trend of improvement in both BCSS and OS. After adjusting for multiple factors in their analysis, these improvements were not statistically significant. As the study population in their article was from before 2009 and did not use more precise methods such as propensity score matching to balance the differences between groups, our study findings provide a better confirmation of this observation.

Although molecular subtype has not been recommended to guide the usage of PMRT, this issue has still been discussed in several studies. Few studies analyzed the different roles of PMRT for older patients on the basis of HER2/neu status and molecular subtypes. The DBCCG 82b and 82c trials analyzed the response to PMRT on different subtypes for patients with PMRT. It showed significant overall survival improvement after receiving PMRT was found in the HR+/HER-2- subtype patients, while not found in the HR-/HER-2 + and triple-negative subtype [23]. Another study for patients with T1-2N1 tumors found that HER2 positive patients (including Luminal B and HER2 enriched subtype) did not improve survival, with only a marginal advantage of overall survival observed for the HR+/ HER-2 + group [24]. Our study displayed the benefit from PRMT for HR+/HER-2- and HR-/HER-2- subtype on both BCSS and OS. Interestingly, elderly patients with HER-2 + tumors did not significantly BCSS benefit from PMRT, with only a marginal survival advantage was shown for the HR+/ HER-2 + subtype in patients with T1-2N1 tumor. Indeed, some literature reported that overexpressed HER-2 with receipt of RT have an increased recurrence risk than HER-2 negative subtype [25]. These results may be due to individual radioresistance associated with multiple molecular mechanisms in the HER-2 positive subtype [26].

A key clinical challenge is to determine specific elderly patients who are more likely to avoid PMRT. Based on the results of the univariable and multivariable analysis in the non-PMRT cohort, we confirmed its independent prognostic factors (age, race, marital status, histology grade, T stage, N stage, ER status, PR status, and given chemotherapy). Then we developed a prognostic nomogram to predict 1-, 3-, and 5-years OS in the non-PMRT cohort. C-index and calibration curves demonstrated its accuracy and discrimination. X-tile helped us to stratify the entire cohort into different risk groups by the optimal cut-off values. Results found that PMRT did not improve overall survival in the low-risk group, while a substantial survival difference is shown in the moderate- and high-risk groups. To the best of our knowledge, this is the first study to build up a nomogram predicting the effect of PMRT in elderly breast cancer patients based on large sample size.

This study has several limitations: (1) The data of neoadjuvant chemotheray, endocrine therapy and targeted therapy was over permission in the SEER database. Also, it does not contain data about the locoregional recurrence rate, which was a predictor widely used to reflect the local tumor control by radiotherapy; (2) A notable limitation of our study is the lack of data on comorbidities within the SEER database, which may impact the interpretation of our results. The absence of comorbidity data may lead to an underestimation or overestimation of the true effect of post-mastectomy radiotherapy on survival in our population, as comorbidities can influence patients’ overall health and prognosis; (3) The retrospective nature of this study may lead to selection bias, although PSM has been introduced to minimize baseline differences between the two groups; thus, we need further prospective trials to validate our findings. One prospective randomized trial, the SUPREMO trial, will be reported in 2023, which randomized patients with T1-2N1, T3N0, or T2N0 to be treated with or without postmastectomy radiotherapy [27].

In conclusion, our study demonstrates that post-mastectomy radiotherapy has a definite role in improving survival for females with elderly breast cancer. After a comprehensive assessment of the side effects and the quality of life, the omission of PMRT could be considered in patients with T1-2N1 breast cancer.

### Electronic supplementary material

Below is the link to the electronic supplementary material.

Supplementary Material 1

Supplementary Material 2

## Data Availability

The datasets generated and/or analyzed during the current study are available in the Surveillance, Epidemiology, and End Results Program (SEER) repository (https://seer.cancer.gov/data/). Any further information about the data can be obtained from the corresponding author.

## Abbreviations

PMRT: post-mastectomy radiotherapy
PSM: Propensity score matching
BCSS: breast cause-specific survival
OS: overall survival
NCCN: National Comprehensive Cancer Network
ER: estrogen receptor
PR: progesterone receptor
HER-2: human epidermal growth factor receptor 2
SMD: standardized mean difference
LN: lymph node
SLNB: sentinel lymph node biopsy
ALND: axillary lymph node dissection
