# Correction: The trade-off of post-mastectomy radiotherapy usage for the breast cancer patients aged 70 years or older: a study based on SEER database

**DOI:** 10.1186/s12877-023-04400-4

**Published:** 2023-11-28

**Authors:** Jingyi Lin, Shiping Luo, Jie Zhang, Chuangui Song

**Affiliations:** 1 Department of Breast Surgery, Fujian Medical University Union Hospital, No.29, Xin Quan Road, Gulou District, 350001 Fuzhou, Fujian Province China; 2 Department of General Surgery, Fujian Medical University Union Hospital, 350001 Fuzhou, Fujian Province China; 3 Breast Cancer Institute, Fujian Medical University, 350001 Fuzhou, Fujian Province China

**BMC Geriatrics (2023) 23:625**

10.1186/s12877-023-04341-y

After publication of this article [1], the authors reported that in this article Lin Jingyi and Luo Shiping should have been denoted as equally contributing authors.

The original article [1] has been corrected.
