# Supplementary material for: The trade-off of post-mastectomy radiotherapy usage for the breast cancer patients aged 70 years or older: a study based on SEER database
Source: BMC Geriatr. 2023 Oct 6;23:625. doi: 10.1186/s12877-023-04341-y (PMC10557241; doi:10.1186/s12877-023-04341-y)
Supplement: Supplementary file 1 — Supplementary Material 1 [file 12877_2023_4341_MOESM1_ESM.docx]

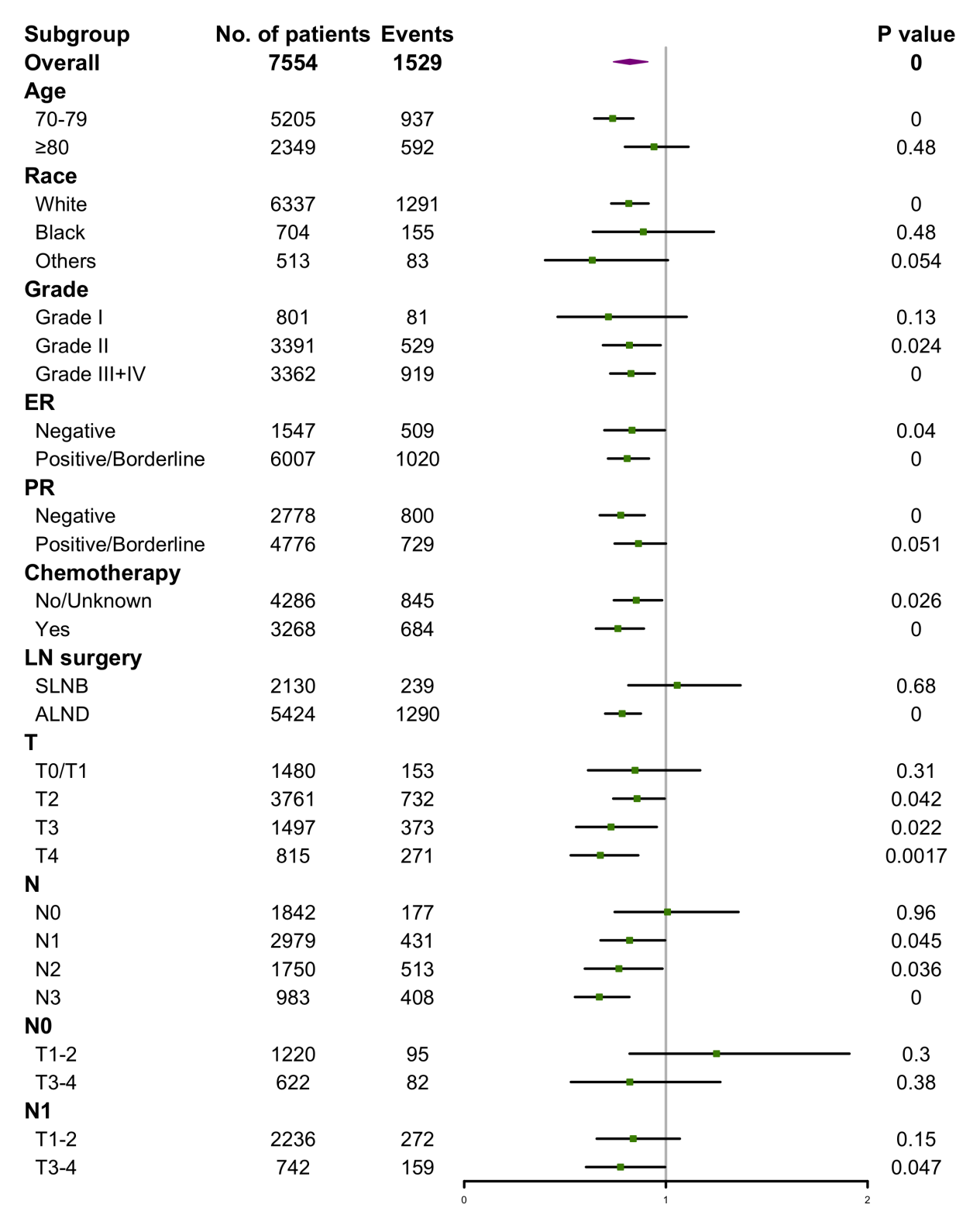


**Supplementary Fig.1** Forest plot of Fine and Gray analysis for BCSD in matched patients. PMRT: post-mastectomy radiotherapy; ER: estrogen receptor; PR: progesterone receptor; LN: lymph node; SLNB=sentinel lymph node biopsy; ALND=axillary lymph node dissection
