# Supplementary material for: The trade-off of post-mastectomy radiotherapy usage for the breast cancer patients aged 70 years or older: a study based on SEER database
Source: BMC Geriatr. 2023 Oct 6;23:625. doi: 10.1186/s12877-023-04341-y (PMC10557241; doi:10.1186/s12877-023-04341-y)
Supplement: Supplementary file 2 — Supplementary Material 2 [file 12877_2023_4341_MOESM2_ESM.docx]

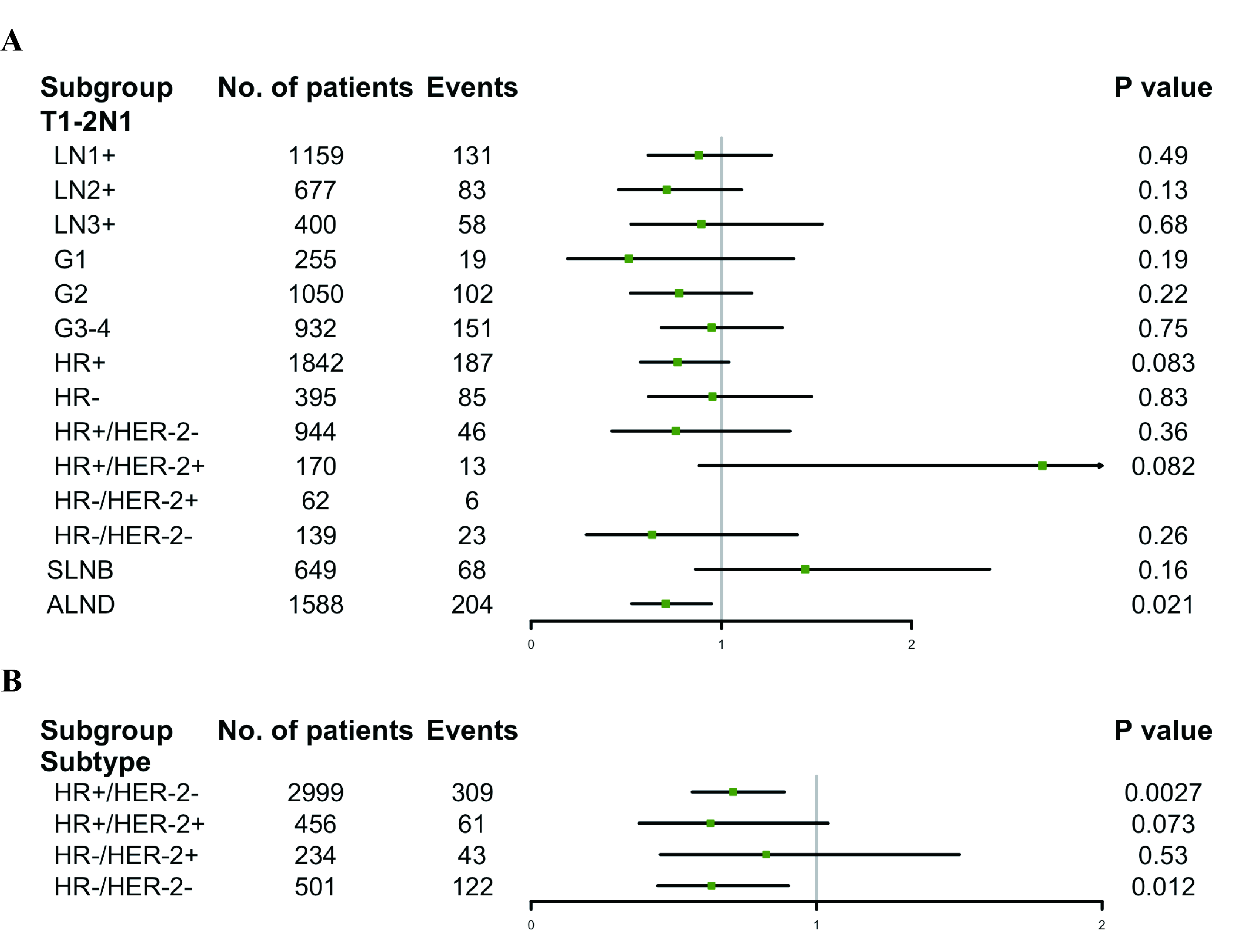


**Supplementary Fig.2** Forest plots of Fine and Gray analysis for BCSD in matched patients with (A) T1-2N1 breast cancer and (B) in patients rematched by known subtype. LN: lymph node; G: Grade; HR: hormone receptor; HER-2: human epidermal growth factor receptor 2; SLNB=sentinel lymph node biopsy; ALND=axillary lymph node dissection
